# Clotrimazole Loaded Ufosomes for Topical Delivery: Formulation Development and In-Vitro Studies

**DOI:** 10.3390/molecules24173139

**Published:** 2019-08-29

**Authors:** Pradeep Kumar Bolla, Carlos A. Meraz, Victor A. Rodriguez, Isaac Deaguero, Mahima Singh, Venkata Kashyap Yellepeddi, Jwala Renukuntla

**Affiliations:** 1 Department of Biomedical Engineering, College of Engineering, The University of Texas at El Paso, 500 W University Ave, El Paso, TX 79968, USA; 2 Department of Pharmaceutical Sciences, University of the Sciences in Philadelphia, Philadelphia, PA 19104, USA; 3 Division of Clinical Pharmacology, Department of Pediatrics, University of Utah, Salt Lake City, UT 84112, USA; 4 Department of Pharmaceutics and Pharmaceutical Chemistry, College of Pharmacy, University of Utah, Salt Lake City, UT 84112, USA; 5 Department of Basic Pharmaceutical Sciences, Fred Wilson School of Pharmacy, High Point University, High Point, NC 27240, USA

**Keywords:** ufosomes, clotrimazole, topical, cholesterol, tape-stripping, permegear flow-through diffusion cells, sodium oleate

## Abstract

Global incidence of superficial fungal infections caused by dermatophytes is high and affects around 40 million people. It is the fourth most common cause of infection. Clotrimazole, a broad spectrum imidazole antifungal agent is widely used to treat fungal infections. Conventional topical formulations of clotrimazole are intended to treat infections by effective penetration of drugs into the stratum corneum. However, drawbacks such as poor dermal bioavailability, poor penetration, and variable drug levels limit the efficiency. The present study aims to load clotrimazole into ufosomes and evaluate its topical bioavailability. Clotrimazole loaded ufosomes were prepared using cholesterol and sodium oleate by thin film hydration technique and evaluated for size, polydispersity index, and entrapment efficiency to obtain optimized formulation. Optimized formulation was characterized using scanning electron microscopy (SEM), X-ray diffraction (XRD), and differential scanning calorimetry (DSC). Skin diffusion studies and tape-stripping were performed using human skin to determine the amount of clotrimazole accumulated in different layers of the skin. Results showed that the optimized formulation had vesicle size <250 nm with ~84% entrapment efficiency. XRD and DSC confirmed the entrapment of clotrimazole into ufosomes. No permeation was observed through the skin up to 24 h following the permeation studies. Tape-stripping revealed that ufosomes led to accumulation of more clotrimazole in the skin compared to marketed formulation (Perrigo). Overall, results revealed the capability of ufosomes in improving the skin bioavailability of clotrimazole.

## 1. Introduction

Global incidence of fungal infections has been on a significant rise since 1980s affecting approximately one billion people and are the primary cause of death in one million pateints annually [1]. The majority of fungal infections are opportunistic or secondary infections in immunocompromised patients with severe diseases such as acquired immune deficiency syndrome (AIDS), tuberculosis, cancer, and chronic obstructive pulmonary disease. The surge in the incidence fungal infections in recent times is due to the increased use of surgical and invasive procedures, immunosuppressants, and antibiotics [2]. Most commonly reported fungal infections are superficial in nature affecting skin, hair, nails, and mucosa however, systemic fungal infections are also reported [1,3]. Superficial fungal infections caused by dermatophytes affect around 40 million people in developed and underdeveloped countries and is the fourth most common cause of infection [3,4]. Common fungal pathogens responsible for fungal infections include *Candida*, *Aspergillus*, *Cryptococcus*, *Scedosporium*, *Zygomycetes*, and other species [1,3]. Various antifungal agents are used for the treatment of fungal infections which include azoles (triazoles and imidazoles), allylamines, polyene antibiotics, echinocandins, griseofulvin, and others [2,3].

Candidiasis, also referred to as yeast infection is the most common fungal superficial infection caused by the Candida species. *Candida albicans* is a ubiquitous fungal pathogen responsible for 50% of the Candida infections and is usually colonized in skin, vagina, mouth, and intestinal tract [2,5,6]. Other candida pathogens include *Candida krusei*, *Candida glabrata*, *Candida lusitaniae*, *Candida tropicalis*, and *Candida Parapsilosis* [7]. Topical formulations are intended to treat local infections on the topmost layer of the skin by effectively penetrating the drugs into the stratum corneum, thus destroying the fungi or the causative organism. Advantages associated with topical formulations include limited systemic bioavailability of the drug, which reduces the systemic adverse effects, potential self-medication, increased patient compliance, and targeted or localized therapy. However, topical preparations have disadvantages such as poor dermal or ungual bioavailability, poor penetration into the stratum corneum, variable drug levels at the site of infection, greasiness or stickiness of ointments and creams, skin irritation, allergic reactions, and uncontrolled evaporation of drugs from the preparation [3,8,9,10,11]. Therefore, there is a need for novel topical formulations to address the problems associated with the current existing formulations. Recently, formulation scientists have explored nanoparticle-based drug delivery systems to improve topical formulations. This is done by delivering active drugs precisely to the infection site while enhancing skin penetration, reducing irritation, and increasing the sustained effect [12]. Several novel drug delivery systems have been formulated to encapsulate antifungal agents and improve their efficacy. Some of them include microemulsions, nanoemulsions, niosomes, dendrimers, solid lipid nanoparticles, liposomes, ethosomes, lipid nanoparticles, and polymeric nanoparticles [3].

Clotrimazole, a broad spectrum less toxic imidazole antifungal agent is widely used to treat Candidiasis. It acts by inhibiting cytochrome 14α-demethylase enzyme of the fungal cells responsible for cell wall synthesis [13]. Chemically, clotrimazole is 1-((2-chlorophenyl) diphenylmethyl)-1H-imidazole, insoluble in water (0.49 mg/L) with Log P of 6.1 and pKa 6.7 [3,14,15]. It is the first oral azole approved for fungal infections; however, it is not used as an oral agent due to its limited oral absorption and systemic toxicity. Currently, clotrimazole is available as conventional topical formulations such as cream (Lotrimin AF and Gyne-Lotrimin), solution (Lotrimin AF), and lotion (Lotrimin AF) [3]. Topical bioavailability of clotrimazole is very low ranging from 0.5% to 10% due to its poor aqueous solubility [14]. Therefore, clotrimazole must be loaded into a suitable drug delivery system to enhance its topical bioavailability at the infection site. It is reported in the literature that clotrimazole has been loaded into various novel drug delivery systems such as nanogels, microemulsions, solid lipid nanoparticles, nanocapsules, ethosomes, three-dimensionally structured hybrid vesicles, and liposomes [5,6,13,16,17]. On the other hand, vesicular drug delivery systems have become more popular in recent times due to their advantages such as prolonged drug release, improved drug penetration, targeted delivery to the site of infection, and improved physical stability. Very recently, Csongradi et al., reported the use of ufosomes as a potential topical drug delivery vehicle. They loaded roxithromycin, a poorly water soluble antibiotic into ufosomes and evaluated its release and skin distribution. Results showed that significant amount of roxithromycin accumulation in the epidermis-dermis layer of the skin with no permeation across the skin layers [18]. Ufosomes are lipid based vesicular drug delivery systems otherwise called unsaturated fatty acid liposomes. They are colloidal suspensions of fatty acids and ionized soaps which can form lipid bilayers and entrap active lipophilic drugs. Loading of clotrimazole into ufosomes can result in enhanced skin penetration due to the lipophilic nature of the vesicles. Ufosomes being fatty acid vesicles, could interact with the stratum corneum and enhance the topical bioavailability of clotrimazole [18,19]. Moreover, preparation ufosomes is economical as the fatty acids are inexpensive compared to other lipids [19]. Therefore, the present study aims to prepare and characterize clotrimazole loaded ufosomes using cholesterol and sodium oleate. Clotrimazole ufosomes were prepared by thin film hydration technique. Effect of drug to excipient ratio on size and entrapment efficiency was studied to optimize the formulation. Further, the formulation was characterized, and skin diffusion studies were performed using human skin to determine the amount of clotrimazole accumulated in different layers of the skin.

## 2. Results and Discussion

### 2.1. Optimization of Clotrimazole Loaded Ufosomes

Optimization studies were aimed to obtain smaller sized ufosomes with high entrapment efficiency. For this, different ratios of clotrimazole, cholesterol and sodium oleate were screened for vesicle size and entrapment efficiency. As most of the clotrimazole topical formulations available commercially are of 1%, the concentration of clotrimazole in all the formulations was constant at 1% *w/v* in all the formulations. The ratio of non-ionized fatty acids and ionized fatty acids determines the stability of ufosomes [19]. Therefore, effect of six different ratios of cholesterol and sodium oleate on vesicle size, polydispersity index, zeta potential, and entrapment efficiency was studied to obtain the optimized formulation. Cholesterol (neutral fatty acid) and sodium oleate (ionized fatty acid) were selected for formulation of ufosomes as they are widely used in lipid based topical formulations. Moreover, they are approved by United States Food and Drug Admininstration (USFDA) as inactive ingredients [20]. Cholesterol, a naturally available unsaturated steroid is capable of forming phospholipid bilayers which entrap hydrophobic drugs. In addition, cholesterol enhanced the stability and permeability of the vesicles [21,22]. It has been used in the preparation of various formulations which include liposomes, ufosomes, topical ointments and creams, transfersomes, niosomes, secosomes, and solid lipid nanoparticles [18,23,24,25,26,27]. Sodium oleate is a salt of unsaturated fatty acid (oleic acid), used as a permeation enhancer in topical formulations and is a generally recognized as safe (GRAS) chemical [18,28,29]. As ufosomes are formed in narrow pH range of 7–9, phosphate buffered saline (PBS) 7.4 was used for hydration. Any differences in the pH of hydration medium will lead to the formation of oil droplets or precipitates (pH < 7) or soluble micelles (pH > 9) in the formulation [30].

#### 2.1.1. Determination of Size, Polydispersity Index, and Zeta Potential

**Figure 1:**
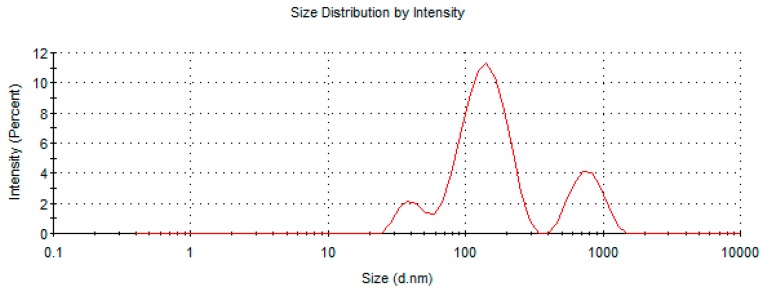
Size distribution curve of Ufo_6.

Table 1 summarizes the size, polydispersity index, and zeta potential of ufosomes prepared using different ratios of cholesterol, sodium oleate, and clotrimazole. Results showed that an increase in the concentration of sodium oleate and cholesterol led to the formation of ufosomes with smaller vesicle sizes. Ufosomes with particle sizes < 300 nm were obtained with Ufo_6 (1:2:2 ratio) (clotrimazole (50 mg), sodium oleate (100 mg) and cholesterol (100 mg)). However, all the formulations were polydisperse in nature with a polydispersity index ranging from 0.4 to 0.7. Size distribution analysis of all the formulations is provided in Table 2. Ufo_6 formulation showed trimodal size distribution indicating the presence of vesicles with different sizes. Analysis of size distribution curves showed that the vesicle size was < 200 nm for the majority of the vesicles (Figure 1). Decrease in the vesicle size with increase in cholesterol could be attributed to higher packing densities and stability of vesicles with increased cholesterol concentration [27]. Similar findings were obtained when diclofenac was loaded into cholesterol vesicles (diclosomes) [31]. As reported in the literature, ZP values for all the ufosomes was high ranging from −73.7 to −101 mV indicating high stability. An increase in the sodium oleate (ionized soap of fatty acid) concentration resulted in the shift of ZP to higher negative values.

#### 2.1.2. Determination of Entrapment Efficiency

**Figure 2:**
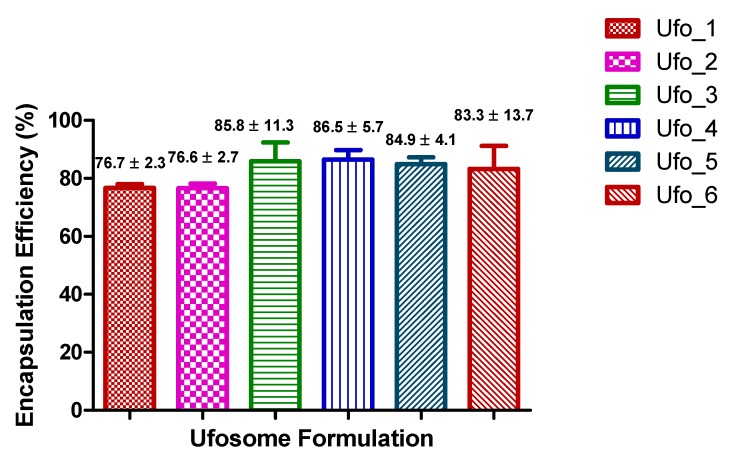
Summary of entrapment efficiency results of ufosomes. Data are presented as mean ± SD (*n* = 3).

Overall, all formulations exhibited high clotrimazole entrapment with entrapment efficiency ranging from ~76% to ~87% indicating ufosomes as ideal carrier for entrapment of lipophilic drugs (Figure 2). Ufosomes contain fatty acids which are oriented in the form of a bilayer with hydrophobic tails towards the interior resulting in greater entrapment of drugs [19]. Ufo_6 formulation had high entrapment efficiency of ~99% for one sample among the triplicates analyzed (high standard deviation). Although, there was no significant difference between the formulations, a trend of greater entrapment was observed with increase in the drug lipid ratio. This could be attributed to higher rigidity of the ufosomal membrane leading to greater drug retention [22]. Additionally, high entrapment efficiency at lower drug to lipid ratio could be attributed to the presence of the enough lipid for entrapment of clotrimazole [32].

Based on the dynamic light scattering (DLS) and entrapment efficiency results, Ufo_6 formulation was chosen as the optimized formulation for further studies due to small vesicle size and high entrapment efficiency.

### 2.2. Surface Morphology

**Figure 3:**
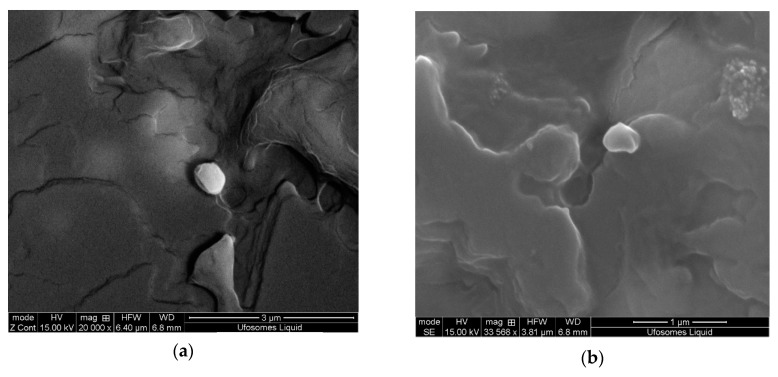
Scanning electron microscopy (SEM) images of clotrimazole loaded ufosomes (Ufo_6).

Scanning electron microscopy (SEM) is one of the most widely used techniques to study the surface morphology of nano and microparticles. This technique uses electron beam as a probe to acquire high resolution images of the particles, whereas DLS provide the hydrodynamic radius of the particles. SEM images of the ufosomes are provided in Figure 3. The images revealed that the ufosomes were roughly spherical with smooth surfaces. Vesicle size observed with SEM was larger compared to the sizes obtained from DLS. The difference in the sizes could be attributed to loss of water during the air drying process resulting in collapse and fusion of vesicles.

### 2.3. X-ray Diffraction 

**Figure 4:**
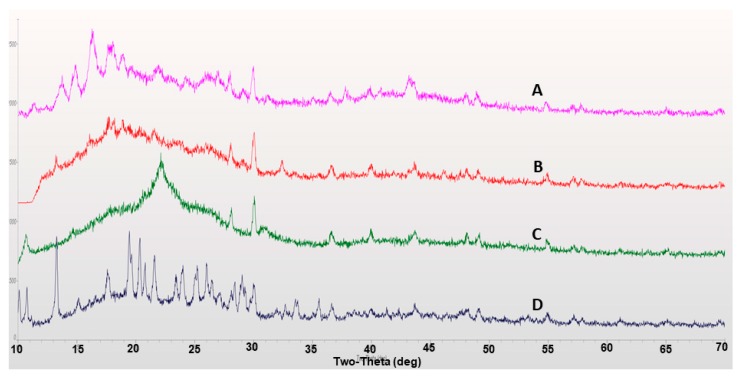
X-ray diffraction (XRD) diffractograms of (**A**) cholesterol, (**B**) clotrimazole loaded ufosomes, (**C**) sodium oleate, and (**D**) clotrimazole.

X-ray diffraction (XRD) studies were performed to study the polymorphic changes of compounds used in the formulation of ufosomes. Crystalline nature of the compounds effects important properties such as stability, solubility, and bioavailability. Amorphous forms of drug molecules are characterized with higher solubilities and increased bioavailability [33,34]. XRD diffractograms for clotrimazole, cholesterol, sodium oleate and clotrimazole loaded ufosomes are provided in Figure 4. Results showed characteristic peaks at 10.8°, 13.2° (high intensity), 19.4°, 20.3°, 21.5°, 24.0°, 25.3°, and 26.2° confirming the crystalline nature of clotrimazole (Figure 4D). In addition, characteristic peaks were recorded for cholesterol (15.3°, 15.8°, and 30°) (Figure 4A) and sodium oleate (30.3°) (Figure 4C). However, XRD results of clotrimazole loaded ufosomes showed the absence of characteristic peaks of clotrimazole (Figure 4B). This confirms the entrapment of clotrimazole into ufosomes and transition of clotrimazole from crystalline to amorphous forms. Similar results were observed in other reports studying the entrapment of drug molecules into lipid-based drug delivery systems [2,32,35,36].

### 2.4. Differential Scanning Calorimetry

**Figure 5:**
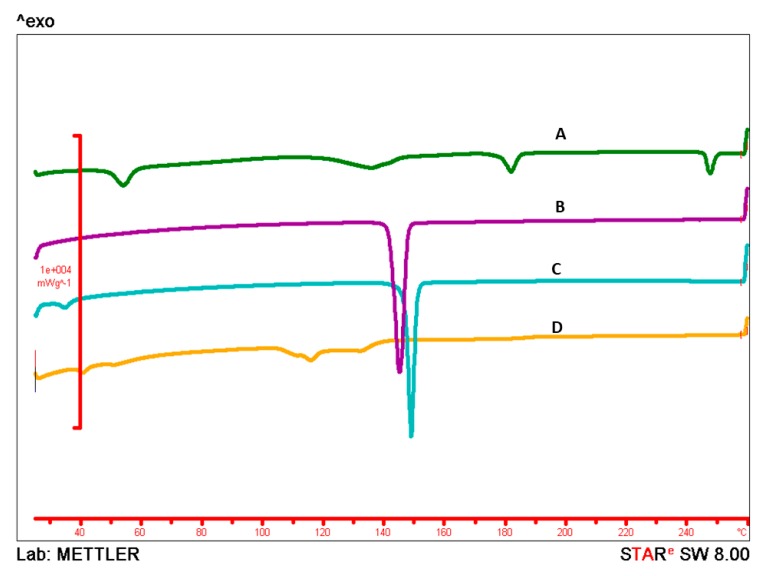
Differential scanning calorimetry (DSC) Thermograms of (**A**) sodium oleate, (**B**) clotrimazole, (**C**) cholesterol, and (**D**) clotrimazole loaded ufosomes. Sharp endothermic peaks in thermograms (**B**,**C**) indicates the melting points of clotrimazole and cholesterol at 147.63 °C and 143.98 °C, respectively. No peak related to the drug was found in clotrimazole loaded ufosomes (**D**).

Differential scanning calorimetry (DSC) is a widely used technique to understand the melting and recrystallization behavior of drug molecules. It is a thermo-analytical technique, that determines the thermodynamic properties of materials by providing information about the polymorphic changes when subjected to a controlled heat flux [37,38]. Thermal behavior of clotrimazole, cholesterol, sodium oleate, and clotrimazole loaded ufosomes is provided in Figure 5. DSC thermogram of clotrimazole showed a characteristic endothermic peak at 143.98 °C. In addition, cholesterol had a melting point of 147.63 °C. No specific thermal behavior was observed for sodium oleate and clotrimazole loaded ufosomes. Disappearance of characteristic endothermic peak of clotrimazole in lyophilized ufosomes confirm the entrapment and transformation from crystalline to amorphous form. DSC thermograms also confirm the internal arrangement of the drug in the vesicles [39]. Clotrimazole is entrapped in cholesterol bilayer and carboxylic groups of sodium oleate are on the surface of vesicles. Overall, DSC results complemented the XRD results.

### 2.5. In-Vitro Permeation Studies

#### 2.5.1. Skin Diffusion Studies

In formulation research and development, in vitro permeation studies are conducted to predict skin permeation of topical and transdermal formulations [40,41]. We used flow-through cells for continuous flow of receptor fluid to maintain sink conditions. Moreover, this type of system is more suitable to simulate in-vivo conditions and preferred for several drug molecules [42]. For our experiments, 24-h permeation studies were conducted on human skin for ufosome suspension and ufosomes in hydroxy propyl methyl cellulose (HPMC) gels (1% and 2%) with blanks as control. For comparison, permeation studies were also conducted for marketed Clotrimazole 1% cream (Perrigo) composed of benzyl alcohol, cetostearyl alcohol, cetyl esters wax, octyldodecanol, polysorbate 60, and sorbitan monostearate. Sampling intervals were 1, 2, 3, 4, 5, 6, 7, 8, 12, 16, 20, and 24 h. Results showed that clotrimazole was not permeated through the skin up to 24 h following the permeation studies from all the tested formulations. Similar results were observed in reports published in literature with clotrimazole microemulsions [43], clotrimazole loaded three-dimensionally-structured hybrid vesicles [17], and roxithromycin ufosomes [18]. As most of the fungal infections are localized on the surface of the skin, systemic bioavailability of clotrimazole is not required. Therefore, ufosomes could be a potential carrier for topical delivery of clotrimazole.

#### 2.5.2. Skin Retention Study (Tape-Stripping)

**Figure 6:**
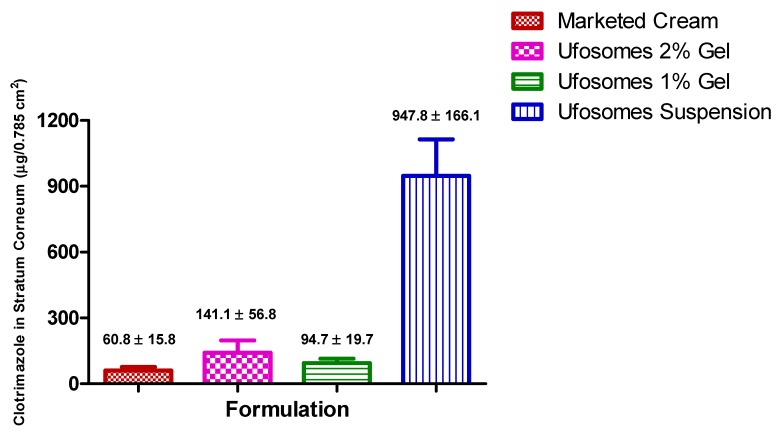
Amount of clotrimazole accumulated in stratum corneum-epidermis layers of skin. Data are represented as mean ± SEM (*n* = 3).

**Figure 7:**
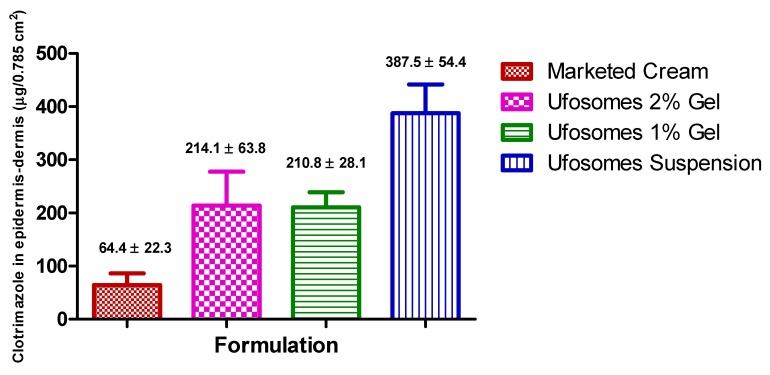
Amount of clotrimazole accumulated in epidermis-dermis layers of skin. Data are represented as mean ± SEM (*n* = 3).

Tape-stripping experiments were performed to determine the penetration of clotrimazole into stratum-corneum, epidermis, and dermis of the skin. Results showed that clotrimazole accumulation in stratum corneum-epidermis and epidermis-dermis layers was significantly higher with ufosomes suspension compared to ufosomes gel and marketed cream (*p* < 0.05). Ufosome gels showed higher levels of clotrimazole in the skin, however, they were not significantly higher compared to the marketed cream (*p* > 0.05). These findings were consistent with other studies where entrapment of drugs into lipid vesicles enhanced the topical bioavailability of drugs. Enhancement of topical bioavailability could be attributed to simultaneous mechanisms such as (i) increased solubility due to transformation of clotrimazole from crystalline to amorphous form, (ii) penetration enhancing property of cholesterol and sodium oleate, and (iii) high interaction of lipids could modify the structure of stratum corneum and (iv) enhanced thermodynamic activity [18,44]. The amount of clotrimazole accumulated in stratum corneum-epidermis and epidermis-dermis layers is provided in Figure 6 and Figure 7, respectively. Compared with marketed formulation, there was ~16-times, ~2.3-times, ~1.5-times enhanced penetration of clotrimazole into stratum corneum with ufosomes suspension, ufosome 1% gel, and ufosomes 2% gel, respectively (Figure 6). Whereas, the amount of clotrimazole accumulated in the epidermis-dermis layer was ~6-fold, ~3.3-fold, and ~3.2-fold higher with ufosomes suspension, ufosome 1% gel, and ufosomes 2% gel, compared to marketed formulation (Figure 7). Overall, topical diffusion studies revealed enhanced penetration and targeted delivery of clotrimazole only to the superior layers of the skin. This proves that ufosomes could be effective in enhancing the required drug concentrations at the target cutaneous tissues.

## 3. Materials and Methods

### 3.1. Materials

Clotrimazole and cholesterol were purchased from Alfa Aesar (Ward Hill, MA, USA). Sodium oleate was purchased from TCI America (Portland, OR, USA). Methanol (ACS grade), methanol (HPLC grade), PBS (pH 7.4), and chloroform were procured from Fisher Chemicals (Fair Lawn, NJ, USA). Hydroxy propyl methyl cellulose (HPMC) (MW: 86,000, viscosity 4000 cP at 2% solution) was obtained from Acros Organics (Fair Lawn, NJ, USA). Cadaver skin for permeation and skin diffusion studies was obtained from Zen-Bio Inc., (Research Triangle Park, NC, USA). Marketed Clotrimazole 1% cream (Perrigo) was purchased from local pharmacy store in El Paso, TX, USA. Deionized water (resistivity of 18.2 MΩ) used for all experiments was obtained from in-house Milli-Q^®^ IQ 7000 Ultrapure Water System (EMD Millipore, Bedford, MA, USA).

### 3.2. Methods

#### 3.2.1. Preparation of Ufosomes

Clotrimazole loaded ufosomes were prepared using thin film hydration method reported in the literature with slight modifications [18,19,45]. Briefly, all the components of the vesicles (clotrimazole, sodium oleate and cholesterol) were dissolved in 10 mL of chloroform-methanol solution (1:2). Clear solution obtained was transferred to a beaker and kept on a magnetic stirrer for complete evaporation of the solvents until a thin film was formed. The thin film was then hydrated with 5 mL of PBS (pH 7.4) for 2 h. The formed vesicular dispersion was sonicated for 5 min to obtain ufosomes with uniform sizes. Optimization of the formulation was performed by screening six different ratios of clotrimazole, sodium oleate and cholesterol for vesicle size and entrapment efficiency. Ufosomal gels for permeation studies were prepared by adding 100 mg (2% *w/v*) and 50 mg (1% *w/v*) of HPMC to the optimized vesicular dispersion.

#### 3.2.2. Determination of Vesicle Size, Polydispersity Index, and Zeta Potential

Dynamic light scattering (DLS) technique applying photon correlation spectroscopy was used to measure vesicle size and polydispersity index. Zeta potential was determined by measuring the electrophoretic mobility. Ufosomal dispersion samples (100 µL) were diluted in 10 mL double distilled de-ionized water and measurements were obtained using Malvern Zetasizer (Nano ZS90, Malvern, Worcestershire, UK) at 25 °C. All the experiments were performed in triplicate.

#### 3.2.3. Clotrimazole Quantification by HPLC

The concentrations of clotrimazole in all the samples obtained during the analysis was determined using Waters Alliance e2695 HPLC with 2998 photodiode array detector and Empower 3.0 software. Clotrimazole separation was carried out on a reverse phase-C_18_ column (Phenomenex^®^; 250 × 4.6 mm; 5 µm particle size) at 25 °C under isocratic conditions. Mobile phase was methanol-water (90:10 v/v) at a flowrate of 1 mL/min. A sample of 20 µL was injected and the analyte was monitored at 229 nm for 10 min. Retention time of clotrimazole was 5.5 min. All standard samples were prepared with methanol and filtered through 0.45 µm filter before injection [13].

#### 3.2.4. Determination of Entrapment Efficiency

Entrapment efficiency of the formulations was determined using ultra-centrifugation method [30]. In brief, the vesicular dispersions were transferred to tubes and centrifuged at 15,000 rpm for 4 h at 4 °C (Beckman Ultracentrifuge). The supernatant was discarded to remove the unentrapped drug in the formulation. The lipid precipitate obtained was then mixed with methanol, bath sonicated for 30 min, and kept overnight in shaking water bath (25 °C; 100 rpm) for complete extraction of entrapped clotrimazole. The resultant solution was centrifuged at 15,000 rpm for 30 min at 4 °C to separate methanol and lipid layer, if any. After centrifugation, supernatant was diluted appropriately and the concentration of entrapped clotrimazole was determined using HPLC. Entrapment efficiency was calculated using the following formula.

Entrapment efficiency (%EE) = (Amount of clotrimazole remained in vesicles)/(Initial amount of clotrimazole) × 100.(1)

#### 3.2.5. Surface Morphology

Quanta 600F scanning electron microscope with a high-resolution field emission source (ThermoFisher Scientific, Hillsboro, OR) was used to study surface morphology of ufosomes. Prior to imaging, samples were dispersed in methanol and the mixture was drop casted onto a piece of silicon wafer (5 × 5 mm) and fixed with double sided conductive tape. Further, samples were air dried and coated with gold using a gold sputter (Gatan 682 Precision Etching and Coating System (PECS) (Gatan, Inc., Pleasanton, CA, USA)). High resolution images of the ufosomes were visualized under high vacuum at an accelerated voltage of 20 keV.

#### 3.2.6. XRD Analysis

XRD analysis was carried out using Rigaku Miniflex X-Ray Diffractometer (Rigaku Corporation, Tokyo, Japan). A double-sided adhesive tape was applied over the sample holder and powdered (lyophilized) samples were poured onto the sample holder using a thin spatula. Intensity of diffracted beam was analyzed in 2θ range between 10° and 70°. All samples were analyzed using JADE software.

#### 3.2.7. DSC

DSC analysis was performed for lyophilized ufosomes, clotrimazole, sodium oleate and cholesterol using DSC822e (Mettler Toledo) instrument. Samples (5–10 mg) were weighed in 40 µL aluminum pans and hermetically sealed using a crimping device. An empty aluminum pan was used as a reference standard on the other side. Nitrogen was used as purge gas during the analysis at a flow rate of 20 mL/min. Samples were held at 0 °C isotherm for 5 min then heated at 10 °C/min to 260 °C (from 0–260 °C, 260–0 °C, 0–260 °C, and finally 260–0 °C). All the thermograms were recorded and analyzed using STARe software.

#### 3.2.8. In-Vitro Permeation Studies

##### Preparation of Skin

Skin penetration studies were performed using a human cadaver defatted skin from the abdominal region of a Caucasian male (ZenBio Inc., Research Triangle Park, NC, USA, Lot#SKIN122117C). Skin acquired from the skin bank was stored in −20 °C freezer until needed. For permeation studies, the skin samples were thawed at 4 °C for 24 h. On the day of permeation study, the skin samples were removed from the 4 °C and allowed to equilibrate at room temperature for 15 min. After equilibration, skin was shaved to remove any hair. Further, the skin was rehydrated in 150 mL of PBS (pH 7.4) for 30 min at room temperature [46,47]. Immediately after rehydration, the full thickness skin was appropriately cut to size of the PermeGear^®^ in-line cells, 1.77 cm^2^, and mounted between the donor and receptor compartments for skin diffusion studies [48].

##### Automated Flow through Diffusion Cells

**Figure 8:**
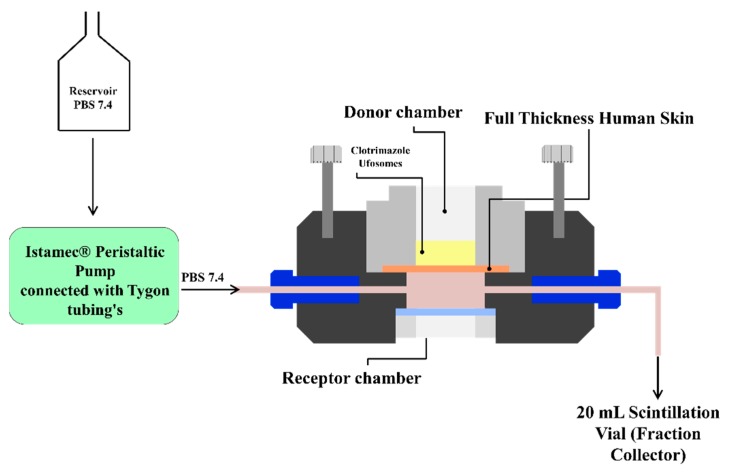
Automated flow through cells.

In vitro permeation studies were performed using PermeGear^®^ ILC-07 automated system (PermeGear, Riegelsville, PA, USA) incorporated with seven in-line flow-through diffusion cells, made of Kel-F. Diffusion cells contain a donor and receptor chambers clamped using threaded rods with adjustable locking nuts. Inlet and outlet ports of the receptor chamber (254 µL receptor chamber volume) were connected to the Tygon tubings having 1/4-28 HPLC fittings and all cells were placed in cell warmer connected to a Julabo BC4 circulating water bath (Seelbach, Germany) to maintain the temperature at 37 °C. All the cells were connected to a multi-channel peristaltic pump^®^ IPC (Ismatec, Zurich, Switzerland) which draws receptor solution from a reservoir (Figure 8). The diameter of the diffusional area was 1 cm (total diffusional area: 0.785 cm^2^). Full thickness skin (epidermis facing donor compartment) was mounted in the cells between the donor and receptor chambers and clamped using the adjustable locking nuts. Formulations were placed in the donor chamber and the receptor fluid (PBS pH 7.4) was pumped at a flow rate of 4 mL/h through each cell. Receptor fluid was collected in the receptor vials of 20 mL capacity at pre-determined time intervals up to 24 h [42,49]. The amount of clotrimazole permeated through the skin was determined by analyzing the samples using HPLC method described in the earlier sections.

##### Tape Stripping

Tape stripping method reported in the literature was employed to determine the amount of clotrimazole accumulated in stratum corneum-epidermis and epidermis-dermis layers of the skin. After completion of skin permeation studies, the skin samples mounted were carefully removed from the cells and placed on a flat surface to view the diffusion area. The excess formulations on the skin surface was removed by gently dabbing the skin using soft tissue. Stratum corneum and some parts of epidermis layers was separated using 3M Scotch^®^ Magic tape™. Pre-cut tapes were pressed onto the skin with the thumb and removed immediately using forceps to remove stratum-corneum (first strip was discarded). An average of 10 strips were required to remove the stratum corneum completely. The remaining epidermis-dermis layers of the skin was cut into tiny pieces using clean surgical scissors. All the strips (except the first one) and skin pieces were transferred to a conical tube with 10 mL methanol and clotrimazole was extracted by sonicating in an ultrasonic water bath for 30 min and left to stand overnight at 4 °C. After extraction, the resulting solution was centrifuged for 30 min at 15,000 rpm and the drug content in the supernatant was analyzed using HPLC [18,44,50,51].

#### 3.2.9. Statistical Analysis

Statistical analysis was performed using GraphPad Prism^®^ software (version 5.0, San Diego, CA, USA). Data are expressed as mean ± SD for DLS and entrapment efficiency results, and mean ± SEM for skin diffusion results. One-way ANOVA followed by followed by Bonferroni’s posttest was applied to determine statistical significance. A *p*-value of < 0.05 was considered as statistically significant.

## 4. Conclusions

Entrapment of hydrophobic drugs into lipid-based vesicles can improve the topical bioavailability. In the present study, we successfully prepared clotrimazole loaded ufosomes using cholesterol and sodium oleate. Optimized formulation with vesicle size < 250 nm and high entrapment efficiency was obtained with 1:2:2 ratio of clotrimazole (50 mg), cholesterol (100 mg), and sodium oleate (100 mg). DSC and XRD results confirmed the successful entrapment of clotrimazole into ufosomes. Spherical morphology of ufosomes was confirmed by SEM. In-vitro permeation studies using human skin revealed that clotrimazole did not permeate through the skin. Topical diffusion studies (tape-stripping) confirmed that ufosomes suspension significantly increased the accumulation of highly lipophilic drug (clotrimazole) into the viable epidermis and dermis as compared to the ufosomes gels and commercially available cream. Thus, this study proves that ufosomes could be a potential carrier to enhance the topical bioavailability and targeted delivery of drugs. However, the results from this study are preliminary and should be further confirmed with in-depth formulation development, stability, and pre-clinical studies before clinical applications.

## Figures and Tables

**Table 1:** Summary of vesicle diameter, polydispersity index and zeta potential results of ufosomes. Data are presented as mean ± SD (*n* = 3).

Formulation	Clotrimazole:Cholesterol:Sodium Oleate	Vesicle Diameter (nm)	Polydispersity Index	Zeta Potential (mV)
Ufo_1	1:0.5:1	1177 ± 156	0.414 ± 0.164	−74 ± 3
Ufo_2	1:1:0.5	848 ± 239	0.638 ± 0.166	−74 ± 3
Ufo_3	1:1:1	432 ± 140	0.583 ± 0.069	−74 ± 5
Ufo_4	1:2:1	374 ± 67	0.589 ± 0.064	−75 ± 7
Ufo_5	1:1:2	752 ± 179	0.702 ± 0.067	−101 ± 5
Ufo_6	1:2:2	234 ± 59	0.581 ± 0.132	−98 ± 3

**Table 2:** Summary of size distribution analysis of ufosomes.

Formulation	Average Vesicle Diameter (nm)	Size Distribution (nm) (Mean ± SD)	Intensity (%)
Ufo_1	1282	1207 ± 342	93.7
		128 ± 27	5.2
		5560	1.1
Ufo_2	894	947 ± 191	82.7
		96 ± 17	17.3
Ufo_3	493	396 ± 80	72.5
		68 ± 12	27.5
Ufo_4	367	546 ± 140	65
		86 ± 21	35
Ufo_5	720	808 ± 167	59.6
		123 ± 28	40.4
Ufo_6	207	144 ± 50	71.6
		782 ± 194	19.4
		42 ± 9	9
